# fMRI as an outcome measure in clinical trials: A systematic review in clinicaltrials.gov

**DOI:** 10.1002/brb3.2089

**Published:** 2021-03-04

**Authors:** Alaleh Sadraee, Martin Paulus, Hamed Ekhtiari

**Affiliations:** ^1^ Institute for Cognitive Science Studies Tehran Iran; ^2^ Iranian National Center for Addiction Studies Tehran University of Medical Sciences Tehran Iran; ^3^ Laureate Institute for Brain Research Tulsa OK USA

**Keywords:** biomarker, clinical trial, Clinicaltrials.gov, fMRI, RDoC

## Abstract

**Introduction:**

More than one‐thousand trials with functional magnetic resonance imaging (fMRI) as an outcome measure were registered in clinicaltrials.gov at the time of writing this article. However, 93% of these registered trials are still not completed with published results and there is no picture available about methodological dimensions of these ongoing trials with fMRI as an outcome measure.

**Methods:**

We collected trials that use fMRI as an outcome measure in the ClinicalTrials.gov registry on 13 October 2018 and reviewed each trial's record entry. Eligible trials’ characteristics were extracted and summarized.

**Results:**

In total, 1,386 clinical trials were identified that reported fMRI in their outcome measures with fMRI as the only primary outcome in 33% of them. 82% of fMRI trials were started after 2011. The most frequent intervention was drug (pharmacological intervention) (29%). 57% of trials had parallel assignment design and 20% were designed for cross‐over assignment. For task‐based fMRI, cognitive systems (46%) based on Research Domain Criteria (RDoC) was the most frequent domain of tasks. Less than one‐third of trials (28%) registered at least one region of interest for their analysis. Food cue reactivity task, pain perception task, n‐back task, and monetary incentive delay task were recruited in more than 25 registered trials.

**Conclusion:**

The number of fMRI trials (fMRI as an outcome measure) with both task and rest protocols is growing rapidly. Our study suggests a growing need for harmonization and standardized checklists on both methods and analysis for preregistration of fMRI‐based outcomes in clinical trials.

## Graphical abstract

The number of preregistered clinical trials with fMRI as an outcome measure (fMRI clinical trials) is increasing rapidly. The characteristics of fMRI clinical trials were reviewed.

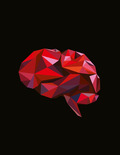

## 1 INTRODUCTION

fMRI is one of the most powerful and dominant imaging techniques in the living human brain by now that have entered a variety of branches of today's clinical research to apply new advances in clinical practice. Speaking of clinical trials, fMRI’s involvement in different domains is so acknowledged that in the current NIH case studies regarding their 2014 definition of clinical trials, there are specific case studies for fMRI in 6 variations trying to elucidate the distinction of the different roles of fMRI in clinical trials as a clinical measurement tool and also as an intervention (National Institutes of Health, 2018, case #18” a‐f”).

In our expectation, the role of fMRI in clinical trials would hopefully be as a biomarker by definition, that is, “a characteristic that is objectively measured and evaluated as an indicator of normal biological processes, pathogenic processes, or pharmacologic responses to a therapeutic intervention” (Biomarkers Definitions Working Group, et al., 2001). To be more specific, fMRI is supposed to be used in order to facilitate the development of new interventions by objectively monitoring functional changes in the brain, hypothetically targeted by the interventions. For example, changes in specific brain activity after taking pharmacological agents are detected by fMRI to evaluate the effect of treatment in early phases in drug trials. In this regard, fMRI would be promising for accelerating the development of new treatment; In drug development for mental health disorders, the urgent need for new treatment was addressed by an NIMH program, Fast‐Fail trials (FAST), utilizing target‐engagement biomarkers including fMRI as one of the potential functional biomarker. fMRI also might play an exploratory role in clinical trials to providing a more objective evidence base for submission to regulators, for example, in later phases of drug trials (Carmichael et al., 2018). In this study, we examined fMRI as an outcome measure, but speaking more broadly, by NIH definition of intervention—manipulation of the subject or subject's environment for the purpose of modifying one or more health‐related biomedical or behavioral processes and/or endpoints—it seems that fMRI can also be considered as an intervention in clinical trials in certain conditions (e.g., presurgical mapping) (National Institutes of Health, 2018, case #18e).

Nonetheless, for fMRI to become a powerful biomarker in clinical trials, still some technical and logistical measures need to be taken in order to make the results reliable and valid. There is a high‐range of false‐positive results in fMRI activations (Wager et al., 2009) and the vast choice of analysis is one of the main reasons (Poldrack et al., 2017); Carp showed that 6,912 unique analysis pipelines are possible for one single dataset which leads to 34,560 significance maps that vary in activation strength, location, and extent (Carp, 2012). Registration of fMRI methodology before the study or preregistration which restricts “research's degree of freedom”—a term coined by Simmons (Simmons et al., 2011), meaning the flexibility of researcher at methodological level— is one of the suggested logistical measures to deal with false‐positive results (Button et al., 2013; Munafò et al., 2017; Poldrack et al., 2017; Carmichael et al., 2018). Clinical trials’ protocol registration in publicly available clinical trial registries is mandatory by laws and policies (https://clinicaltrials.gov/ct2/about‐site/history). ClinicalTrials.gov is the largest clinical trials registry with over 200,000 registered studies. The registry was first established to make possible the public access to clinical trials for participation purposes, and as the need for transparency and reproducibility in science emerges, it extended its goals to also serve as a registry for better monitoring and tracking of clinical trials (Zarin & Keselman, 2007). As time passed, the site tightened its rules and modified the protocol registration's required information for more accurate and complete registration. However, for fMRI in clinical trials, the best practices for preregistration needs to be well‐defined through a detailed checklist. If following that checklist becomes obligatory by policymakers and granting agencies as a module in the protocol registration in formal clinical trial registries, like clinicaltrials.gov, hopefully, it will result in more valid and replicable fMRI outcomes in clinical trials in long term.

In this study, we completed a systematic review to investigate the scope of fMRI clinical trials in clinicaltrials.gov. We extracted the available data for characteristics of fMRI clinical trials. For categorizing a large number of tasks in task‐based fMRI trials, we chose NIMH Research Domain Criteria (RDoC) construct definition (https://www.nimh.nih.gov/research‐priorities/rdoc/index.shtml). As a potential preregistration role that clinicaltrials.gov could play in the future for an fMRI‐specific data element, we also explored the extent of available preregistered data in clinicaltrials.gov and provided a few recommendations.

## 2 METHOD

### 2.1 Search strategy and study selection

We searched clinicaltirals.gov on 13/10/2018 for potential clinical trials that used task‐based or resting‐state fMRI as their outcome measure by using the search term fMRI. Clinicaltrials.gov is a registry that includes more than clinical trials (or interventional studies). Hence, the search was then restricted to clinical trials by applying one of the potential search filters “study type: Interventional”. For further analysis, we downloaded the results in a tab‐separated value format and transferred it to an excel table to fix the dataset for later analysis. The dataset encompassed eligible trials’ protocol mandatory required information (data elements) which was submitted to the clinicaltrials.gov registry by the sponsor or principal investigator at the start of the study. In order to include completed or ongoing clinical trials, we excluded Suspended, Terminated, Withdrawn, and Unknown study states. We also excluded the following studies by manually reviewing each trial's record page:

1. Trials that the outcome measures were recording data of real‐time neurofeedback fMRI to reduce methodological complexities
2. Trials with empty (not recorded) outcome measures
3. Trials that didn't report fMRI in their outcome measures either as a consequence of incomplete reporting or in other cases, for example, if fMRI was used as a part of an intervention in the trial, like “brain surgery based on presurgical planning with fMRI”
4. Explicitly stated Not BOLD fMRI trials, for example, perfusion fMRI
5. Nonrelevant trials that appeared in our result as a result of the similarity of keywords

**FIGURE 1:**
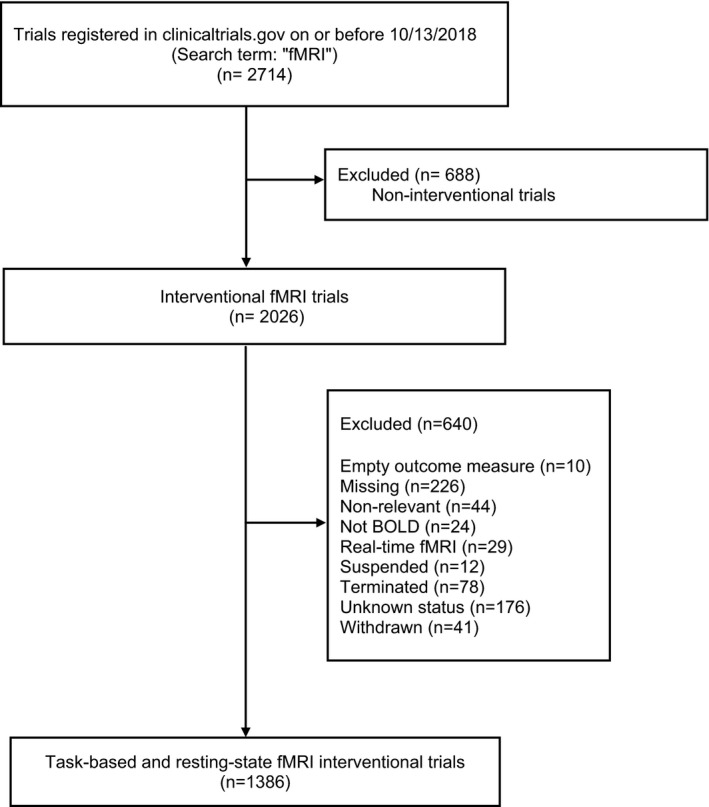
Inclusion flowchart of fMRI trials registered in clinicaltrials.gov on or before 10/13/2018

Trials’ selection process is outlined in the flow diagram (Figure 1). To follow the best practices in reporting, we applied relevant “Preferred Reporting Items for Systematic Reviews and Meta‐analyses” PRISMA guidelines (Moher et al., 2010). See Checklist S1 for PRISMA checklist of items.

### 2.2 Data collection

The following variables are the general characteristics that were available in the dataset:

Recruitment, Start Date, Primary Completion Duration (Year), Location, Funding Source, Gender, Age, Intervention, Primary Purpose, Phase, Intervention Model, Masking, Allocation, Enrollment.

The authors then manually reviewed each trial's record page for extracting fMRI variables:

fMRI as an Outcome Measure, fMRI Type, Task Name, and Reported Region of Interests.

### 2.3 Definition and classification of variables

Details of all data elements’ definitions in the XML file are available at the ClinicalTrials.gov (https://prsinfo.clinicaltrials.gov/definitions.html).

#### 2.3.1 fMRI as an outcome measure

It defines whether fMRI serves as the “only primary outcome measure” (for clinical trials that have no other primary outcome measures except for fMRI), “one of the primary outcome measures”, “one of the secondary outcome measures” (but not in the primary outcome measures), or “other outcome measures” (but not in the primary or secondary outcome measures) of the trial. It is better to mention that primary outcome measures’ groups also encompass trials that use fMRI as secondary in addition to primary outcome measures, and we didn't categorize trials that use fMRI in both primary and secondary outcome measures in an individual group due to the importance of primary compared with secondary outcome.

#### 2.3.2 fMRI method

fMRI method used as an outcome measure is categorized on three main groups of “Resting‐state fMRI”, “Task‐based fMRI”, “Resting‐state fMRI‐Task‐based fMRI”; Studies that didn't mention their fMRI method explicitly were labeled as “Not specified” with two exceptions: If the study mentioned the stimuli (e.g., brain response to visual food cues) or an action (e.g., moving hand), we included them in task‐based fMRI.

#### 2.3.3 Task name

In order to be able to categorize the tasks, the task name provided by authors’ manipulation (e.g., “brain response to visual food cues” were considered as “food cue reactivity task”). We categorized tasks within the framework of RDoC. For the sake of clarity, the “cognitive systems” domain is broken into its sub‐constructs (attention, perception, working memory, declarative memory, language, and cognitive control). Because of the ambiguity in names of the tasks, the ones related to each RDoC domain are classified into three groups of “Well‐ specified”, “Partially specified”, and “Not specified (Table S2).

#### 2.3.4 Reported region of interests

We recorded data on whether trials reported at least one major region in which the activation will be examined or have a hypothesis included the activation of at least one region; for resting‐state fMRI, we also included common networks (e.g., default mode network (DMN)) as reported regions of interest. As this section was a part of a bigger plan to examine trials from preregistration aspect (see the introduction and discussion), we excluded studies with results for this part. Furthermore, fMRI trials that included drug (pharmacological intervention) as the only intervention or one of the interventions were reported separately as well.

### 2.4 Statistical analysis

All characteristic data are summarized and analyzed using Excel 2016. Categorical variables are reported as frequencies and percentages.

## 3 RESULT

The ClinicalTrials.gov registry includes 2026 entries for interventional studies with fMRI as a keyword. Among these, we identified 1,386 completed (46%) or ongoing clinical trials (54%) that used BOLD fMRI as an outcome measure. The reasons for exclusion of 640 interventional trials are listed in Figure 1. Among completed trials, 84% didn't report their results yet.

The most frequent intervention was drug (28.7%) (Table 1). All 396 clinical trials with drug as the intervention (pharmacological interventions) and 96 clinical trials with multiple interventions including drug were reported exclusively as “drug intervention” (494 trials) in Tables 1, 2, 3, 4, 5. In 33% of trials, fMRI was the only primary outcome (Table 2). 50% of trials had less than 50 participants and 6% more than 200. 82% of fMRI trials were started after 2011. 57% of trials had parallel assignment design and 20% were designed for cross over assignment (Table 3). More details on the characteristics of trials can be found in Table 2 and Table 3.

**TABLE 1:** Intervention of fMRI trials registered in clinicaltrials.gov on or before 10/13/2018

Intervention	Number of trials (1,386)	Percent
Drug	398	28.7
Behavioral	292	21.1
Device	221	15.9
Multiple interventions (Included drug)	96	6.9
Multiple interventions (Not‐included drug)	95	6.9
Other	179	12.9
Dietary supplement	38	2.7
Procedure	45	3.2
Biological	9	0.6
Radiation	6	0.4
Diagnostic test	6	0.4
Genetic	1	0.1

**TABLE 2:** General characteristics of fMRI trials registered in clinicaltrials.gov on or before 10/13/2018

General characteristics	Number of trials		Percent	
	All interventions (1,386)	Drug interventions (494)	All interventions	Drug interventions
fMRI as an outcome measure				
fMRI as the only primary outcome	452	205	32.6	41.5
fMRI as one of the primary outcomes	346	128	25.0	25.9
fMRI as one of the secondary outcomes	497	134	35.9	27.1
Other outcomes	91	27	6.6	5.5
Recruitment				
Not yet recruiting	113	27	8.2	5.5
Recruiting	536	152	38.7	30.8
Enrolling by invitation	17	7	1.2	1.4
Active, not recruiting	79	22	5.7	4.5
Completed with result	100	72	7.2	14.6
Completed without result	541	214	39.0	43.3
Start date				
1998–2002	7	4	0.5	0.8
2003–2006	58	34	4.2	6.9
2007–2010	183	93	13.2	18.8
2011–2014	382	145	27.6	29.4
2015–2018	745	214	53.8	43.3
2019	9	4	0.6	0.8
NR^a^	2	0	0.1	0.0
Primary completion duration (year)^b^				
<1	208	76	15.0	15.4
1–2	649	233	46.8	47.2
3–4	358	118	25.8	23.9
5–6	111	43	8.0	8.7
7 or more	50	17	3.6	3.4
NR	10	7	0.7	1.4
Location				
United States	639	259	46.1	52.4
Europe	415	137	29.9	27.7
Asia	132	43	9.5	8.7
Canada	73	20	5.3	4.0
Other (South America‐Africa‐Australia)	20	5	1.4	1.0
NR	107	30	7.7	6.1
Funding source				
Other	977	284	70.5	57.5
Other/NIH	181	75	13.1	15.2
Other/industry	83	48	6.0	9.7
Industry	63	56	4.5	11.3
Other/U.S. Fed	25	8	1.8	1.6
NIH	25	15	1.8	3.0
U.S. Fed	22	4	1.6	0.8
Other multiple funding sources	10	4	0.7	0.8
Gender				
Both	1,174	379	84.7	76.7
Female	88	37	6.3	7.5
Male	123	77	8.9	15.6
NR	1	1	0.1	0.2
Age^c^				
Adult, older adult	626	172	45.2	34.8
Adult	582	277	42.0	56.1
Child	72	15	5.2	3.0
Child, adult	56	21	4.0	4.3
Older adult	23	3	1.7	0.6
Child, adult, older Adult	27	6	1.9	1.2

^a^ Not Registered (Not‐Reported)

^b^ (Primary Completion date ‐ start date)

^c^ Child (=<18), Adult (18–65), Older Adult (>=65)

**TABLE 3:** Design characteristics of fMRI trials registered in clinicaltrials.gov on or before 10/13/2018

Design	Number of trials		Percent	
	All interventions (1,386)	Drug interventions (494)	All interventions	Drug interventions
Primary purpose				
Treatment	627	210	45.2	42.5
Basic science	433	185	31.2	37.4
Other	93	31	6.7	6.3
Diagnostic	76	28	5.5	5.7
Prevention	56	9	4.0	1.8
Supportive care	34	6	2.5	1.2
Health services research	13	7	0.9	1.4
Device feasibility	5	0	0.4	0.0
Screening	3	1	0.2	0.2
NR	46	17	3.3	3.4
Phases				
Early phase 1	57	40	4.1	8.1
Phase 1	134	114	9.7	23.1
Phase 1‐Phase 2	33	17	2.4	3.4
Phase 2	104	79	7.5	16.0
Phase 2‐Phase 3	20	9	1.4	1.8
Phase 3	26	13	1.9	2.6
Phase 4	100	94	7.2	19.0
Not applicable	912	128	65.8	25.9
Intervention model				
Parallel assignment	793	246	57.2	49.8
Crossover assignment	283	161	20.4	32.6
Single group assignment	259	66	18.7	13.4
Factorial assignment	41	17	3.0	3.4
Sequential assignment	8	3	0.6	0.6
NR	2	1	0.1	0.2
Masking (blinding)				
None (open label)	465	96	33.5	19.4
Single	276	44	19.9	8.9
Double	297	149	21.4	30.2
Triple	177	94	12.8	19.0
Quadruple	163	105	11.8	21.3
NR	8	6	0.6	1.2
Allocation				
Randomized	996	402	71.9	81.4
Nonrandomized	188	47	13.6	9.5
NR	202	45	14.6	9.1
Enrollment				
0–50	689	254	49.7	51.4
51–100	412	143	29.7	28.9
101–200	204	70	14.7	14.2
201–300	53	18	3.8	3.6
301 or more	24	7	1.7	1.4
NR	4	2	0.3	0.4

**TABLE 4:** RDoC domain of task‐based fMRI trials registers in clinicaltrials.gov on or before 10/13/201

RDoC domain		Number of tasks		Percent			
		All trials (963)	Drug trials (431)	All trials		Drug trials	
Negative valence systems		50	29	5.2		6.7	
Positive valence systems		186	99	19.3		23.0	
Negative and/or positive valence systems		38	21	3.9		4.9	
Cognitive Systems	Attention	32	12	3.3	45.6	2.8	39.9
	Perception	98	30	10.2		7.0	
	Declarative memory	61	26	6.3		6.0	
	Language	23	10	2.4		2.3	
	Cognitive control	143	52	14.8		12.1	
	Working memory	82	42	8.5		9.7	
Systems for social processes		99	60	10.3		13.9	
Arousal/Regulatory Systems		1	1	0.1		0.2	
Sensorimotor Systems		52	7	5.4		1.6	
Not specified		98	42	10.2		9.7	

**TABLE 5:** Task classification of task‐based fMRI trials registered in clinicaltrials.gov on or before 10/13/2018 based on RDoC domains for well‐specified tasks with more than 10 repetitions

Task	Number	
	All trials (281)	Drug trials (133)
Food cue reactivity task	49	16
Pain perception task	35	13
*N*‐back task	34	17
Monetary incentive delay task	27	20
Motor task (active, passive movement)	21	6
Go/no‐go task	19	9
Stop signal task	17	8
Smoking cue reactivity task	16	7
Regulation task (emotion)	15	5
Stroop task	13	6
Fear conditioning task	13	9
Drug cue reactivity task	11	7
Alcohol cue reactivity task	11	10

Forty‐two percent of trials registered task‐based fMRI, 19% resting‐state, and 12% of trials registered both as the outcome measures. There were 963 tasks in 753 trials that reported task‐based fMRI as a part of their outcome measures. 20% of trials used more than one task (Table S1 provides details of the number of tasks in these trials). Positive valence systems (19%), cognitive control (15%), perception (10%), and systems for social processes (10%) were the most frequent domains/subdomains of tasks based on RDoC (Table 4). Food cue reactivity task, pain perception task, n‐back task, and monetary incentive delay task were recruited in more than 25 registered trials (Table 5). You can find more details on the fMRI tasks in each domain in Table S2. The trend of use in the most frequently recruited fMRI tasks is also reported in Table 6. We found that 28% of trials have reported at least one region of interest. We also included some of the best pre‐registered data samples of preprocessing and analysis plan that was found in our clinical trials dataset in Table S3. The dataset is also shared in the Supplementary Dataset.

**TABLE 6:** The trend of well‐specified tasks with more than 10 repetitions from 2001–2018

Task Year	2001–2003	2004–2006	2007–2009	2010–2012	2013–2015	2016–2018
Cue reactivity task (food) (48)^a^	0	2	5	12	12	17
Pain perception task (35)	0	4	4	9	7	11
*N*‐back task (34)	0	2	3	7	10	12
Monetary incentive delay (MID) task (27)	0	0	0	5	13	9
Motor task (active, passive movement) (21)	0	2	2	3	6	8
Go/no‐go task (19)	0	0	2	4	4	9
Cue reactivity task (smoking) (16)	0	1	3	4	4	4
Regulation task (emotion) (15)	1	0	1	3	5	5
Stroop task (13)	0	0	4	5	1	3
Fear conditioning task (13)	0	0	0	3	3	7
Cue reactivity task (drug) (11)	0	1	0	3	2	5
Cue reactivity task (alcohol) (11)	0	1	1	1	1	7
Task‐based fMRI (962)*	5	33	104	150	280	390

^a^ One of the studies’ start date is missed.

## 4 DISCUSSION

In order to develop an understanding of fMRI usage in clinical trials as an outcome measure, we systematically reviewed the fundamental characteristics of eligible trials registered in the ClinicalTrials.gov before 2019. During the last 20 years, fMRI as an outcome measure has grown rapidly such that about half of the total trials with fMRI as an outcome measure were registered after the year 2016. 54% of fMRI trials are still ongoing and 84% of the completed trials didn't report their result yet so we still do not have a good methodological representation of these ongoing or completed trials in the published literature. This is the first report to give an overview of the current status of preregistered fMRI‐based outcome measures in clinical trials based on the available data on clinicaltrials.gov. In about one‐third of the included trials in this systematic review, fMRI was the only primary outcome measure and the “Primary Purpose” of half of the trials was reported as “treatment”. 29% of the trials examined a drug intervention. 70% of trials had randomized allocation. Triple and Quadruple blindness overall made up 25 percent of trials. Cognitive systems (46%) based on RDoC were the most frequent domain of tasks, followed by positive valence systems (19%), systems for social processing (10%), and sensorimotor systems (5%). Emotional processing tasks, cue reactivity tasks, pain perception tasks, N‐back task, emotional faces processing task, motor tasks, and Go/no‐go task were the most frequent tasks that used in these trials (please see Supplementary Dataset 2 for more details). fMRI statistical analysis details in registered trials are scarce and less than one‐third of trials registered at least one region of interest for their analysis.

We reviewed all registered materials for each trial to extract fMRI‐specific information that might be reported in different sections such as outcome measures, study arms, detailed description. In about 25 percent of trials, the resting‐state or task‐based fMRI types were not specified. In about 10 percent of task‐based fMRI trials, there was not any clear indication (even of task‐domain). Task's name as well as reporting model was varied according to the following categories: (a) Conventional name mentioned explicitly (e.g., monetary incentive delay task) (b) Task was introduced in a general way (e.g., decision‐making task, cognitive task) (c) The procedure was indicated (e.g., listen to baby's cry, exposure to auditory and visual food cues, luminous stimulation) (d) Task's name was not mentioned but the task's details were described, and (e) Evaluation of brain response to stimuli in study implicated the task (e.g., brain activity in response to noxious stimuli, as assessed by fMRI). We organized the data to be consistent and placed them in tabular format by categorizing the task domains according to the RDoC definition.

In an effort to search for preregistered data of fMRI specification, we had a plan to collect details of registered fMRI information including data design, data acquisition, data preprocessing, and analysis plan, but the minute amount of available data and the lack of similarity in reporting style posted restrictions on this procedure so that we were unable to take our preregistration classification any further. Even for basic details like regions of interest (ROI), less than one‐third of trials registered at least one ROI for their analysis.

At this point, there is sufficient evidence that higher standards of preregistration information are needed to help transparency and reproducibility of research, which would lead to bias reduction. Overall, as Munafò et al. (2017) have mentioned, study design, primary outcome(s), and analysis plans are what should be prespecified for a study in the strongest form of preregistration. There are also some previous efforts to recommend essentials for an fMRI study preregistration; editors of “Neuroimaging: Clinical” referred to the potentials for prediction in clinical neuroimaging studies and importance of preregistration of ROIs as a specific suggestion against SHARKing. “This is common in clinical trials and there is no reason that strong predictions cannot be defined in clinical neuroimaging studies. Preregistration of ROIs may be particularly useful as it guards against SHARKing “(Roiser et al., 2016). Poldrack and colleagues (2017) have indicated “planned sample size, specific analysis tools to be used, specification of predicted outcomes, and definition of any specific ROIs or localizer strategies that will be used for analysis” as the details of an fMRI study that should be preregistered. Particularly in clinical trials—to extend Carmichael et al., 2018 statement is helpful (even though it is initially recommended for drug development trials): “fMRI methodology should be held to the same standard as other clinical endpoints, namely, methods must be prespecified and fixed for the duration of the study. This prespecification should include a thorough description of task design and implementation, image acquisition and quality control, data preprocessing, ROI definition, model estimation, and endpoint calculation (Schwarz et al. 2011, 2011a, b)”.

As we reported in Table S3, there are many examples of good practices in preregistration of methods and analysis details among the trials we have studied. As an example, in a clinical trial registered as NCT02218736 “Change in Ventral Striatal Activation Occurring in Anticipation of Reward During the Monetary Incentive Delay Task Measured by fMRI (Time Frame: baseline, Week 8)” is considered as the only primary outcome measure. This outcome is defined as “Establish POC (Proof of Concept) for KOR (Kappa Opioid Receptor) antagonism by evaluating the impact of CERC‐501 relative to Placebo on reward‐related neural circuitry in terms of ventral striatal activation during anticipation of reward during the Monetary Incentive Delay Task. Evaluation by fMRI (Functional magnetic resonance imaging). The BOLD (Blood Oxygen Level‐Dependent) score on the Z‐scale represents how far the actual measured intensity is from the expected in the template. A score of 0 would correspond to the mean/median, a score of 1.65 would represent the 90‐percentile, −1.65 the 10‐percentile, and so on, according to a standard normal distribution”. In this preregistration, researchers have provided details on the methods and analysis for this fMRI‐based outcome measure, however, there are still details needed to increase the replicability of the outcome measure such as task version, task analysis contrast, and many details in the preprocessing, ROI definition and data analysis pipeline. We believe that higher level of reproducibility could be facilitated with a detailed checklist that should be developed in a consensus process (Ekhtiari, et al., 2020). It is true that clinicaltrial.gov website is not technically designed to provide, encourage or fully support this checklist, however, we should mention that there is still an available space in the clinicaltrial.gov website for each trial to provide detailed descriptions up to 32,000 characters, and also there is a possibility for uploading additional documents for preregistration including structured checklists for methodological details.

The current study is the first one that tried to provide an overview of registered trials with fMRI as an outcome measure in clinicaltrials.gov to the best of our knowledge. It should be mentioned that ClinicalTrials.gov is not the sole registry for all clinical trials in the world, so our database is a limited subset of all fMRI clinical trials. The use of fMRI as an outcome measures in clinical trials is growing fast, imposing the requirement for a considerable amount of registration standardization for fMRI‐specific information to make the accumulating data useful for researchers and assure the validation of results. Based on this systematic review, we suggest the following actions:

### 4.1 Recommendation 1

fMRI type (task‐based fMRI versus resting‐state fMRI, and basics of pulse sequence like BOLD EPI) and the detailed description of the task/rest should be provided in a clear and replicable way. Sometimes, even with the label of famous fMRI tasks, investigators recruit various versions with major or minor differences. A consensus on a list of major fMRI tasks with their available codes/stimuli could reduce this variability. While methodological variations in fMRI tasks (if reported carefully in a replicable way) are welcome and can contribute to our better understanding of the variations in the explored phenomenon, the use of identical standardized publically available fMRI tasks in different trials should be encouraged to increase harmonization and data interpretability.

### 4.2 Recommendation 2

Providing official reference checklist of specifically required items for preregistered clinical trials with fMRI as an outcome measure will be important. Committee on Best Practices in Data Analysis and Sharing (COBIDAS) offered a comprehensive reporting checklist for an fMRI study (Nichols et al., 2017). From the authors’ perspective, for a strong form of preregistration, the content of conventional method section of a reported fMRI study should be specified beforehand (registered‐report). Hence, we recommend specifying items in Tables D1–D5 of the COBIDAS reporting checklist for preregistration.

### 4.3 Recommendation 3

Preregistration of fMRI analysis details in a replicable way is a labor‐demanding job. Having a citable list of most acceptable fMRI analysis pipelines within major analysis platforms without wiggling room for further analytics explorations could be very helpful to increase transparency and replicability. Authors could still enjoy exploratory analysis in the trial outcomes, but explicit differentiation between (a) confirmatory analysis based on a well‐defined preregistered analysis pipeline and (b) exploratory analysis should be clearly reported in the publications.

### 4.4 Recommendation 4

Definition of minimum requirements for fMRI outcome preregistration in major platforms like clinicaltrials.gov and then suggestions of the optimum list of items to be registered is required. The expectation for a comprehensive preregistration for fMRI analysis in the first step might be significantly complex for investigators with less experience and can act as a serious barrier.

### 4.5 Recommendation 5

All preregistration efforts should not suppress potentials for innovations in new task design and more efficient fMRI analysis pipeline development. We also support secondary data exploration when it is transparently reported.

Currently, there is a large gap in harmonized collective efforts with shared fMRI protocols and study designs to help accumulate replicable knowledge in the field over time. Meanwhile, there is a growing effort in the neuroimaging community to develop domain‐specific and domain‐general checklists for better quality reporting of fMRI studies (Ekhtiari et al., 2020). We hope this systematic review and its recommendations will help to move one step forward for this endeavor.

## CONFLICT OF INTEREST

None declared.

## AUTHOR CONTRIBUTION

A.S., M.P. and H.E. conceived of the presented idea and H.E. supervised the project. A.S. gathered data, designed the tables and performed the analytic calculations. All authors discussed the results and contributed to the final manuscript.

### Peer Review

The peer review history for this article is available at https://publons.com/publon/10.1002/brb3.2089.

## Supporting information

Supplementary Material

Table S1‐S3

## Data Availability

The dataset is included in the Supplementary Material.

## References

[brb32089-bib-0001] Biomarkers Definitions Working Group , Atkinson, A. J. Jr, Colburn, W. A. , DeGruttola, V. G. , DeMets, D. L. , Downing, G. J. , & Spilker, B. A. (2001). Biomarkers and surrogate endpoints: Preferred definitions and conceptual framework. Clinical Pharmacology & Therapeutics, 69(3), 89–95. 11240971 10.1067/mcp.2001.113989

[brb32089-bib-0002] Button, K. S. , Ioannidis, J. P. , Mokrysz, C. , Nosek, B. A. , Flint, J. , Robinson, E. S. , & Munafò, M. R. (2013). Power failure: Why small sample size undermines the reliability of neuroscience. Nature Reviews Neuroscience, 14(5), 365. 10.1038/nrn3475. 23571845

[brb32089-bib-0003] Carmichael, O. , Schwarz, A. J. , Chatham, C. H. , Scott, D. , Turner, J. A. , Upadhyay, J. , Coimbra, A. , Goodman, J. A. , Baumgartner, R. , English, B. A. , Apolzan, J. W. , Shankapal, P. , & Hawkins, K. R. (2018). The role of fMRI in drug development. Drug Discovery Today, 23(2), 333–348. 10.1016/j.drudis.2017.11.012. 29154758 PMC5931333

[brb32089-bib-0004] Carp, J. (2012). On the plurality of (methodological) worlds: Estimating the analytic flexibility of fMRI experiments. Frontiers in Neuroscience, 6, 149., 10.3389/fnins.2012.00149. 23087605 PMC3468892

[brb32089-bib-0005] Ekhtiari, H. , Zare‐Bidoky, M. , Sangchooli, A. , Janes, A. C. , Kaufman, M. J. , Oliver, J. , & Baldacchino, A. (2020) A methodological checklist for fMRI drug cue reactivity studies: Development and expert consensus. medRxiv, 10, 4304. 10.1038/s41596-021-00649-4 PMC9063851 35121856

[brb32089-bib-0006] Moher, D. , Liberati, A. , Tetzlaff, J. , & Altman, D. G. (2010). Preferred reporting items for systematic reviews and meta‐analyses: The PRISMA statement. Journal of Surgery, 8, 336–341. 10.1016/j.ijsu.2010.02.007. 20171303

[brb32089-bib-0007] Munafò, M. R. , Nosek, B. A. , Bishop, D. V. M. , Button, K. S. , Chambers, C. D. , Percie du Sert, N. , Simonsohn, U. , Wagenmakers, E.‐J. , Ware, J. J. , & Ioannidis, J. P. A. (2017). A manifesto for reproducible science. Nature Human Behaviour, 1(1), 21. 10.1038/s41562-016-0021. PMC7610724 33954258

[brb32089-bib-0008] National Advisory Mental Health Council Workgroup on Tasks and Measures for Research Domain Criteria, 2016National Advisory Mental Health Council Workgroup on Tasks and Measures for Research Domain Criteria (2016). Behavioral assessment methods for RDoC constructs. MD: Bethesda.

[brb32089-bib-0009] National Institutes of Health (2018). NIH definition of clinical trials case studies: Retrieved from. https://grants.nih.gov/policy/clinical‐trials/case‐studies.htm. January 17

[brb32089-bib-0010] Nichols, T. E. , Das, S. , Eickhoff, S. B. , Evans, A. C. , Glatard, T. , Hanke, M. , Kriegeskorte, N. , Milham, M. P. , Poldrack, R. A. , Poline, J.‐B. , Proal, E. , Thirion, B. , Van Essen, D. C. , White, T. , & Yeo, B. T. T. (2017). Best practices in data analysis and sharing in neuroimaging using MRI. Nature Neuroscience, 20(3), 299. 10.1038/nn.4500. 28230846 PMC5685169

[brb32089-bib-0011] Poldrack, R. A. , Baker, C. I. , Durnez, J. , Gorgolewski, K. J. , Matthews, P. M. , Munafò, M. R. , Nichols, T. E. , Poline, J.‐B. , Vul, E. , & Yarkoni, T. (2017). Scanning the horizon: Towards transparent and reproducible neuroimaging research. Nature Reviews Neuroscience, 18(2), 115. 10.1038/nrn.2016.167. 28053326 PMC6910649

[brb32089-bib-0012] Roiser, J. P. , Linden, D. E. , Gorno‐Tempinin, M. L. , Moran, R. J. , Dickerson, B. C. , & Grafton, S. T. (2016). Minimum statistical standards for submissions to Neuroimage. Clinical. Neuroimage: Clinical, 12, 1045. 27995071 10.1016/j.nicl.2016.08.002 PMC5153601

[brb32089-bib-0013] Schwarz, A. J. , Becerra, L. , Upadhyay, J. , Anderson, J. , Baumgartner, R. , Coimbra, A. , Evelhoch, J. , Hargreaves, R. , Robertson, B. , Iyengar, S. , Tauscher, J. , Bleakman, D. , & Borsook, D. (2011). A procedural framework for good imaging practice in pharmacological fMRI studies applied to drug development# 2: Protocol optimization and best practices. Drug Discovery Today, 16(15–16), 671–682. 10.1016/j.drudis.2011.03.011. 21477664

[brb32089-bib-0014] Schwarz, A. J. , Becerra, L. , Upadhyay, J. , Anderson, J. , Baumgartner, R. , Coimbra, A. , Evelhoch, J. , Hargreaves, R. , Robertson, B. , Iyengar, S. , Tauscher, J. , Bleakman, D. , & Borsook, D. (2011a). A procedural framework for good imaging practice in pharmacological fMRI studies applied to drug development# 1: Processes and requirements. Drug Discovery Today, 16(13–14), 583–593. 10.1016/j.drudis.2011.05.006. 21635967

[brb32089-bib-0015] Simmons, J. P. , Nelson, L. D. , & Simonsohn, U. (2011). False‐positive psychology: Undisclosed flexibility in data collection and analysis allows presenting anything as significant. Psychological Science, 22(11), 1359–1366. 10.1177/0956797611417632. 22006061

[brb32089-bib-0016] Wager, T. D. , Lindquist, M. A. , Nichols, T. E. , Kober, H. , & Van Snellenberg, J. X. (2009). Evaluating the consistency and specificity of neuroimaging data using meta‐analysis. NeuroImage, 45(1), S210–S221. 10.1016/j.neuroimage.2008.10.061. 19063980 PMC3318962

[brb32089-bib-0017] Zarin, D. A. , & Keselman, A. (2007). Registering a clinical trial in ClinicalTrials. gov. Chest, 131(3), 909–912. 10.1378/chest.06-2450. 17303677

