# Supplementary material for: fMRI as an outcome measure in clinical trials: A systematic review in clinicaltrials.gov
Source: Brain Behav. 2021 Mar 4;11(5):e02089. doi: 10.1002/brb3.2089 (PMC8119793; doi:10.1002/brb3.2089)
Supplement: Supplementary file 2 — Table S1‐S3 [file BRB3-11-e02089-s001.docx]

**Table S1.** Number of tasks in each of task-based fMRI trials registered in clinicaltrials.gov on or before 10/13/2018.

| Tasks per trials | Number of trials  (753) | Number of tasks  (963) |
| --- | --- | --- |
| 1 | 602 | 602 |
| 2 | 105 | 210 |
| 3 | 36 | 108 |
| 4 | 7 | 28 |
| 5 | 3 | 15 |

**Table S2.** Task classification of task-based fMRI trials registered in clinicaltrials.gov on or before 1 0/13/2018 based on RDoC domains.

| RDoC domain |  | Task | Number  (963) |
| --- | --- | --- | --- |
| Negative valence systems  (50) | Well specified  (31) | Fear conditioning task  Montreal imaging stress task (MIST)  Passive avoidance task  Aggression task  Script-driven imaging paradigm (individual trauma/ neutral-script)  Listening to trauma scripts  Catastrophizing task  Emotional processing task (IAPS: neutral, negative)  Interpretation bias training (threat learning system)  Emotionally-provoking tasks (periods of breathing restriction)  Emotionally-provoking task (presentations of a loud noise)  Response to breathlessness cues  Listen Infant cry stimuli (listen to auditory stimuli: unknown cries, own cries, and tone controls.) | 13  5  2  2  1  1  1  1  1  1  1  1  1 |
|  | Partially specified  (19) | Emotional processing tasks (negative stimuli)  Rumination task  Emotional challenge task | 17  1  1 |
| Positive valence systems  (186) | Well specified  (149) | Food cue reactivity task  Smoking cue reactivity task  Drug cue reactivity task  Alcohol cue reactivity task  Cue reactivity task (mixed-rewarding)  Cue reactivity task (internet (gaming) craving)  Monetary incentive delay task  Food choice task  Delay discounting task  Balloon analog risk task (BART)  Relative reinforcing value of food task  Alcohol food incentive delay task  Response to beverage taste and logo task  Conditional visuo-motor associations task  Effort expenditure for rewards task (EEfRT)  Cups task  Consumption of fruit-flavored drinks in "choice" and "no choice" context  Liking versus wanting for gustatory stimuli  Iowa Gambling Task (IGT)  Food reward paradigm (expectation and receipt of a pleasant tasting reward)  Rewarding effect of men's verbal compliment on women  Reward processing task (erotic vs. neutral pictures of persons)  Food willingness to pay task  Script-driven imagery (binge-eating)  Food infusion with questions about feelings, cravings and mood changes)  Decision making processes triggered by food labels  Speeded cue-behavior association task | 49  16  11  11  3  1  27  4  3  3  2  2  2  2  1  1  1  1  1  1  1  1  1  1  1  1  1 |
|  | Partially specified  (37) | Reward task  Decision making task  Social reward task  Monetary reward task  Gambling task  Anticipating a positive interoceptive stimulus  Food reward task  Outcome expectation task  Prediction error task  Probabilistic classification task  Probabilistic instrumental learning task  Probabilistic reversal learning task  Feedback-based learning task  Reward anticipation task  Reward-emotional coding task  Social reinforcement learning task  Smoking and monetary reward task  Taste reward learning paradigm (a task for prediction error and reward value computation) | 8  6  5  3  2  1  1  1  1  1  1  1  1  1  1  1  1  1 |
| Negative and/or positive valence systems  (38) | Well specified  (4) | Cue reactivity task (mixed-trauma and alcohol)  Pavlovian to Instrumental Transfer (PIT) paradigm (appetitive, aversive, and drug-related Pavlovian cues)  Passive viewing of video clips (half of the video clips will assess analytic thinking, and half empathetic/emotional cognition)  Emotional anticipation task (reactivity to and recovery from pictures of human suffering and flourishing) | 1  1  1  1 |
|  | Partially specified  (34) | Emotion processing tasks  Emotion processing task(IAPS)  Emotional anticipation task | 31  2  1 |
| Attention  (32) | Well specified  (12) | Interoceptive attention task  Rapid visual information processing (RVIP) task  Rapid serial visual presentation (RSVP)  Breath counting task  Dichotic listening paradigm  Posner's task | 6  2  1  1  1  1 |
|  | Partially specified  (11) | Sustained attention task  Attentional control task  Divided attention task  Concentration task  Letting go practice | 4  3  2  1  1 |
|  | Not specified |  | 9 |
| Perception  (98) | Well specified  (86) | Pain perception task  Stimulation task (Somatosensory)  Checkerboard visual stimulation  Biological motion task  Oddball task  Video-guided acupuncture imagery treatment (VGAIT)  Stimulation (active and inactive auricular tVNS)  Stimulation task (pressure is varied in the right ear)  Stimulation task (monaural sound to the ear)  Stimulation task (Optokinetic stimuli)  Stimulation task (visual)  Bite/non-bite task fMRI (brain activity associated with orthodontic pain)  Breath holding fMRI (BH fMRI) paradigm  Breathing pure oxygen (fMRI in pregnant women)  Central breathing control (during the application of an inspiratory resistance)  Gender categorization task (chimeric faces, entire faces, or faces presented in divided visual field)  Gender classification task (face / voice interaction)  Hypnosis induction  Listen to babies’ cry (to discriminate the intensity of the pain mediated by the cries)  Listen to known and unknown music  Listening to sounds in various contexts  Mentally induced pruritus  Motion discrimination task  Music and/or spoken word (language) listening  order judgement task  Pain perception task (observational and testing phase)  Painful task in empathetic/non-empathetic context  Simple detection task (unilateral and bilateral auditory visual stimulus presentations)  Visual imagery task  Stimulation task (olfactory cues of stress and sport)  Taste, temperature and glucose sensing  Target presentations, with or without target jump (saccadic adaptation)  Visual recognition task  Balloon distension of rectum  Tear drainage (in trigeminal neuralgia)  Responses to abdominal stimuli  Signal detection (SD) task  Processing of vowels | 35  8  3  3  3  2  1  1  1  1  1  1  1  1  1  1  1  1  1  1  1  1  1  1  1  1  1  1  1  1  1  1  1  1  1  1  1  1 |
|  | Partially specified  (12) | Stimulation task(visual)  Stimulation task (sensory)  Stimulation task (auditory)  Stimulation task (visual and olfactory stimuli) | 6  3  2  1 |
| Declarative memory  (61) | Well specified  (22) | Familiar-novel task  Face-name association task  Object completion memory task  Face recognition task  Relational and item specific encoding task (RISE)  Retrieval task (naturalistic episodes)  Word List Recognition Task  Word-stem completion task  Visual version of a paired associates learning task  Paired associate visuospatial learning (PAL)  Associative memory task (image-position)  Associative memory task (face-place)  Declarative memory task (retrieval of a previous learned word list)  Emotional memory paradigm  Episodic memory task (pictures of the World War II)  Episodic memory task (scene encoding and recognition)  Emotional prosody recognition  Object location task  Object pattern separation task | 3  2  1  1  1  1  1  1  1  1  1  1  1  1  1  1  1  1  1 |
|  | Partially specified  (23) | Episodic memory task  Associative memory task  Autobiographical memory task  Declarative memory task  Emotional memory paradigm  Reversal Learning task  Recognition memory task  Spatial memory task  Semantic memory task  Learning new environment  Metamemory paradigm  Virtual reality (VR)-based assessment of multiple learning and memory systems | 7  2  2  2  2  2  1  1  1  1  1  1 |
|  | Not specified |  | 16 |
| Language  (23) | Well specified  (16) | Picture naming task  Word production task  Reading comprehension task  Inner speech task (the imagination of speech)  Pseudo-word Rhyming task  Semantic category task  Silent verb generation task  Speaks, sings, and hums  Verbal and nonverbal vocalizations  Verbal processing task (shallow processing/deep processing of words)  Speech discrimination task  Speech identification task | 3  2  2  1  1  1  1  1  1  1  1  1 |
|  | Partially specified  (1) | Singing-related task | 1 |
|  | Not specified |  | 6 |
| Cognitive control  (143) | Well specified  (114) | Go/no-go task  Stop signal task  Regulation task (emotion)  Stroop task  Multi-source interference task (MSIT)  Regulation task (craving)  Flanker task  Continuous performance task (CPT)  Regulation task (pain)  Simple reaction time task  Digit Symbol Substitution test  Oral symbol digit modalities test  Dot-Probe Expectancy Task  Driving tasks (driving simulation)  Egocentric mental transformation  Preparing to overcome prepotency task  Recognition visual reaction time (RVRT) task  Shifted-attention emotion appraisal (SEAT) paradigm  Simon task  Sustained attention to a response test (SART)  Tasks related to tool use  Car following task (measures tactical control of the vehicle)  Road tracking task (measures operational control of the vehicle)  Gap acceptance task (measures strategic control of the vehicle)  Tower of London  Wisconsin card sorting task  Evaluating deductive reasoning problems  Virtual navigation test  Classify pairs of stimuli (objects and pictures) on the basis of how well they "go together"  Decision making task (deciding whether or not single objects would fit into a shoe box)  Attentional switching task which examines set-shifting and reversal-learning component | 19  17  15  13  8  7  6  5  2  1  1  1  1  1  1  1  1  1  1  1  1  1  1  1  1  1  1  1  1  1  1 |
|  | Partially specified  (17) | Response inhibition task  Task-switching task  Cognitive-emotional interference paradigm  Emotion processing task (task of emotional congruence)  Emotional distractor task  Emotional face interference task (EFIT)  Emotional/non-emotional cognitive conflict paradigm  Goal-directed information processing  Cognitive control task (make different responses to different visual stimuli) | 7  3  1  1  1  1  1  1  1 |
|  | Not specified |  | 12 |
| Working memory  (82) | Well specified  (45) | N-back task  Sternberg working memory task  Paced auditory serial addition test (PASAT)  Delayed match to sample task  Dot working memory task  Reading span fMRI task  Operation span task  Rewarded delayed response working memory task  Self-ordered object working memory task (SOWMT) | 34  4  1  1  1  1  1  1  1 |
|  | Partially specified  (3) | Spatial working memory task  Verbal working memory task | 2  1 |
|  | Not specified |  | 34 |
| Systems for social processes  (99) | Well specified  (54) | Social relationship task*  Emotional face matching task  Hariri face matching task  Emotional faces processing task (recognition task)  Cyberball task  Applying social touch  Emotional faces processing task (gender-categorization task)  Deception detection task  Prisoners' dilemma game  Social incentive delay (SID) task  Social feedback task  Penn Emotion Recognition Task  Affect labeling paradigm  Attribution of intention task  Empathy task (geometric shapes)  Eyes task  Faces task (same-sex face vs other-sex face)  Interpreting Communicative Intent  Iterative version of the classical "Trust Study"  Playing a ball-game (measuring perception of emotional facial videos)  Processing of empathy inducing pictures  Theory of Mind task  Trust Game  Self-referential task  self-appraisal task (read about situations and imagine him/herself being self-critical or self-reassuring in these conditions)  Social reward processing paradigm (rate the valence and arousal of emotional pictures in three different conditions: alone, share with a stranger and share with their friend)  Looking at photographs infant faces  Illness awareness task  Friend or foe task (emotional judgement task) | 8  5  4  3  3  3  2  2  2  2  2  1  1  1  1  1  1  1  1  1  1  1  1  1  1  1  1  1  1 |
|  | Partially specified  (34) | Emotional faces processing task  Self-reflection task  Theory of Mind task  Face-object processing task  Behavioral tasks with condition "Social" and condition "Alone"  Manipulation of self-focused processing  Novel "minimally-joint" paradigm  Self-compassion task  Face matching task  Emotion recognition task | 23  2  2  1  1  1  1  1  1  1 |
|  | Not specified |  | 11 |
| Arousal/Regulatory Systems  (1) | Well specified  (1) | Psychomotor vigilance test | 1 |
| Sensorimotor Systems  (52) | Well specified  (38) | Motor task (active, passive movement)  Motor task (imagery)  Bipedal paradigm  Paced motor task  Automated motor sequence or AT (automated task)  Learning motor sequence (motor sequence task)  Finger-to-thumb opposition sequence  Learned single finger-sequencing task  Finger-tapping task  Tactile object manipulation(act-fMRI)  physical stepping with an MRI-compatible stepping machine, or imagined/visualized cycling | 21  8  1  1  1  1  1  1  1  1  1 |
|  | Partially specified  (3) | Visual-motor task  Writing  Motor learning task | 1  1  1 |
|  | Not specified |  | 11 |
| Not specified |  |  | 98 |

* Response to picture of a beloved person (partner, infant, etc.) versus a stranger

**Table S3.** Samples of fMRI pre-registered data in clinicaltirals.gov for Data Pre-processing/ Analysis: We included some of the most completed samples to give a picture to reader of the available fMRI Pre-processing/ Analysis pre-registered information.

| NCT Number | Data Pre-processing/ Analysis |
| --- | --- |
| NCT03191994 | **Title: Impact of an Eight Week Exercise Intervention in Treating Major Depressive Disorder** |
|  | Using fMRI to determine brain activity during an associative memory task. Participants will be scanned on a 3-Tesla MR scanner. Scans will be acquired in the oblique coronal plane of the hippocampus. 416 functional scans will be acquired with a T2*-weighted gradient EPI sequence. Preprocessing will be performed using Statistical Parametric Mapping. General linear model will be performed at the single-subject level and statistical contrasts will be created modeling the hemodynamic response function of correct and incorrect responses. Random effects analysis will be performed using the contrast of t-test of correct > incorrect. Significant clusters from an independent samples t-test for correct>incorrect at baseline will be used to extract contrast beta values for correct>incorrect in pre and post scans. Average beta values will be imported into SPSS and a 2 x 2 repeated measures ANOVA (group x time). |
| NCT02380573 | **Title: Cognitive and Functional Connectivity Effects of Methylene Blue in Healthy Aging, Mild Cognitive Impairment and Alzheimer's Disease** |
|  | Standard fMRI analysis will be analyzed using established fMRI software. Statistical parametric analysis will be performed to generate activation maps. fMRI data will be corrected for multiple comparisons using a false discovery rate (q < 0.05) and threshold for cluster values to conservatively control for type I error. Behavioral data will be analyzed with paired t-test and ANOVA calculations used for group comparison with p < 0.05 (with Bonferroni correction) considered statistically significant. |
| NCT02394054 | **Title: Vasopressin and the Social Brain** |
|  | Changes in neural brain activity, as observed by fMRI, when observing others during social inclusion vs. exclusion [ Time Frame: Between 40-115 minutes post administration]: Whole brain and region of interest (ROI) regression analysis will be used to compare the neural activity of participants in the vasopressin/placebo groups. A design matrix will be created for each participant, modeling activity that is greater during the exclusion portion of the cyberball task (Williams et al., 2012) versus the inclusion portion. A first-level analysis will compare [(friend exclusion>friend inclusion)> (stranger exclusion>stranger inclusion)] for each participant. A second-level group analysis will compare these first-level contrasts between the vasopressin and placebo groups. |
| NCT02770105 | **Title: Theanine and Caffeine on Neurophysiology of Attention** |
|  | RVRT will be compared across the four conditions (theanine, caffeine, TC and placebo) constructing a repeated measure analysis of variance (ANOVA) model using the R-statistical software. Subsequently, pair-wise comparisons of RVRT will be performed between the four conditions, maintaining the family-wise error rate at 0.05. Following sequence of operations will be conducted on the fMRI data using the FMRIB software library (FSL): visual examination, distortion correction, motion correction, slice timing correction, spatial smoothing and statistical analysis. BOLD responses will be compared across the four conditions by constructing ANOVA models at a whole brain level as well as in the regions of anterior cingulate cortex and antero-medial prefrontal cortex. Pair-wise comparisons of BOLD responses will also be conducted between the conditions. RVRT, omissions and errors will be included as covariates in fMRI analysis. |
| NCT03657056 | **Title: Focused Ultrasound Neuromodulation for Treatment of Temporal Lobe Epilepsy** |
|  | Reliability and repeatability of fMRI scanning will be ensured. For the activation related work in the present experiments, centroids of activation will be identified using the canonical general linear model (GLM) methods in common use, thresholding regions using z statistics calculated under the analysis stream embodied in FSL. In comparing signals across trials and stimulus conditions the extent of suprathreshold activity will not be evaluated, but the activation magnitude, essentially the slope of regression ("beta weights") derived from the GLM will be evaluated. The investigators have shown that, by comparison to the threshold extents, this measure is highly reliable and repeatable across not only individual scans, but over multi-day scanning sessions. Additional information regarding fMRI image processing and analysis is provided in Appendix A. |
| NCT01945957 | **Title: Brain Imaging of Intranasal Oxytocin Treatment in Autism** |
|  | fMRI Activation Analysis/Connectivity [ Time Frame: 30, 75 minutes post dose]: Activation data will be analyzed using FEAT within FSL (Oxford University, U.K.). Onset times of events will be used to model BOLD signal responses containing a regressor for each response type convolved with a double-γ function. A priority region of interest will be the VTA and NAc which will be analyzed via anatomically defined ROI's.  Connectivity Analysis: Time series will be extracted from ROIs using FSL Featquery for each participant and averaged separately for each Treatment (OT, placebo) and Trial Type (rewarded, unrewarded) condition (seed and target regions will be functionally defined on the basis of task response). Correlation coefficients will be transformed using a Fisher r-to z transformation. Mean z-transformed values will then be computed across participants and ROI pairs, and then converted back to correlation coefficients. |
| NCT03286387 | **Title: Contextual Associations During Episodic Recall of Everyday or Virtual Reality** |
|  | BOLD cerebral response to contextual recall in medial temporal lobe [ Time Frame: up to 1 week after the end of the encoding session]: After spatial preprocessing of individual series of EPI fMRI images, normalization and smoothing as suggested for SPM analysis, the successful and failed contextual recall will be modeled and convolved with canonical HRF function. The GLM will be estimated according to the algorithm of SPM, the significance threshold will be p<0.05 FWE within the regions of interest. Our hypothesis will be confirmed by activation of medial temporal lobe and prefrontal cortex in contextual recall, congruent contextual cueing and recall of episodes when targets were semantically congruent with the context at encoding. |
| NCT02393443 | **Title: Oxytocin and Learning for Teaching** |
|  | Changes in brain neural activity, observed by fMRI, in response to a reading comprehension task [ Time Frame: Between 40-90 minutes post administration]: Whole brain and region of interest (ROI) regression analysis will be used to compare the neural activity of participants in the tutor/memorizer and oxytocin/placebo groups. A design matrix will be created for each participant, modeling activity that is greater during reading comprehension compared with the grammar control passage. First level analyses will compare the response during the reading comprehension passages relative to the grammar control passages. Second level group analyses will compare the first level contrasts between the tutor vs. memorizer groups as well as the oxytocin vs. placebo groups. In regression analyses we will enter each participant's comprehension test score in the tutor and then memorizer condition as a regressor in a whole-brain and ROI analysis to determine which brain regions were more active during the encoding of the reading comprehension passage compared to the grammar control passage. |
| NCT02132286 | **Title: Serotonin Transporter Genetic Variation and Amygdala Responses to Antidepressant Medications in Major Depression** |
|  | The first level of analysis will be the generation of fMRI maps of brain activity using FMRIB Software Library (FSL), a statistical software package specifically designed for fMRI applications, which uses the general linear model to compute contrast parameter estimates of explanatory variables (in our case, the explanatory variable is face task vs. sensorimotor task) for each pixel in the brain images. The result is an estimate of the response magnitude in a given pixel that surpasses a specified statistical threshold (typically, p = 0.01).  The main variable of interest will be the magnitude of activity within the amydala. For each patient, regions of interest (ROIs) will be drawn manually to encompass the amygdala within each hemisphere with the aid of each patient's anatomical images. Magnitude of activity with the ROIs will be recorded. A generalized linear mixed model will be used to analyze group data with treatment, and time of session (baseline, 1-week, 8-week) as factors into the analysis. As we expect that the sample size of patients with La/La genotype will be smaller in each treatment group, the primary analysis will be done only in MDD patients with S/Lg alleles to prove our hypothesis. To examine the association between changes in amygdala activity and response in depression severity the change in HAM-D for each patient over 8 weeks will be regressed on the corresponding change in amygdala activity. A similar approach will be used to correlate 1-week change in activity with 8-week change in depression severity. The other regions that are functionally or structurally connected with amygdala such as anterior cingulate region (ACC), dorso-lateral prefrontal cortex (DLPFC), dorso-medial prefrontal cortex (DMPFC), and precuneus (PCC) will also be selected for exploratory analysis. |
| NCT02637271 | **Title: Brain Response to Dietary Interventions** |
|  | fMRI analysis will be carried out using a standard mixed effects model implemented in FMRIB Software Library (FSL). Specifically, analysis of fMRI data will be focused on identifying pre-post differences in both the location and relative magnitude of whole brain activation patterns within and between members of each group (TMR vs. TD). Pre-planned comparisons will also be made for specific regions of interest: a) food-reward related areas (e.g. insula, orbito-frontal cortex, anterior cingulate cortex, and amygdala); b) reward calculating regions (e.g. nucleus accumbens and ventral striatum), c) memory and emotion related areas (e.g. hippocampus and amygdala); d) homeostatic circuitry (i.e. hypothalamus) and cortical areas exerting executive control (e.g. pre-frontal cortex). Both structural and functional connectivity between the considered regions of interest will also be modeled and compared between and within groups. Demographic, health, weight history and psychosocial variables may be included as covariates in specific analyses as indicated. |
| NCT02569034 | **Title: Dissociating Components of Anhedonia: A Pilot fMRI Study** |
|  | To quantify brain activity associated with reward "wanting," the investigators will contrast fMRI activation while participants view information about reward probability and choose to perform the easy or hard task with activity during fixation. The investigators will contrast activation during performance feedback and presentation of reward magnitude with activation during fixation to capture brain activity associated with reward "liking." Parameter estimates (ß) will be generated, which indicated relative strength of covariance between the data and the hemodynamic response function (HRF). The investigators will use a three-dimensional spatial contiguity threshold of 20 voxels and a statistical significance threshold of p ≤ .005 to decrease the likelihood of spurious findings. |
| NCT03652012 | **Title: Probing Cortical Excitability and Cognitive Function With TMS** |
|  | The resting-state fMRI data will be preprocessed using a pipeline (http://wiki.biac.duke.edu/biac:analysis:resting_pipeline) and tools in the Oxford Centre for Functional MRI of the Brain Software Library (FSL version 5.0.5, www.fmrib.ox.ac.uk/fsl). The preprocessing steps include slice-time correction, MCFLIRT for motion correction, Brain Extraction Tool (BET) for brain extraction, and FLIRT for normalization to the MNI 152 T1 template (Montreal Neurological Institute, Montreal, Canada). The investigators will regress out signal from white matter and cerebrospinal fluid on the basis of masks created in FSL FAST and smoothed the data with a 5 mm kernel using FSL SUSAN. Temporal band-pass filtering will limit the data to frequencies in the 0.001 to 0.08 Hz band. Following the recommendation of Power et al., the investigators will perform motion scrubbing using a frame-wise displacement threshold of 0.5 and timecourse variance threshold (DVARS) of 0.5% |
| NCT03403998 | **Title: Neck Exercises in Patients With Temporomandibular Disorders** |
|  | To analyze the DTI images, we will use a method similar to Moayedi et al. To analyze the rsfMRI images, we will use the pipeline outlined in Greicius et al.in addition to applying functional connectivity (graphical modelling) as previously used in our lab. Images will be imported into the software library (FSL v. 4.1.8) of the Oxford Centre for Functional MRI of the Brain (FMRIB). Preprocessing will include current and motion artifact correction using the FMRIB Diffusion Toolbox v. 2.0. The DTI images will be processed through 2 different pipelines for 1) voxel-wise analysis and 2) tractography. The preprocessed images will be fit with a diffusion tensor model using DTIFIT in the FDT. We then will calculate voxel-wise values of FA. The rsfMRI images will be analyzed using an ICA approach to isolate the DMN and the sensorimotor network and a functional connectivity approach using graphical modelling to assess the neural networks associated with TMD treatment. |
